# Supplementary material for: The Construction and Exploration of a Comprehensive MicroRNA Centered Regulatory Network in Foxtail Millet (Setaria italica L.)
Source: Front Plant Sci. 2022 May 6;13:848474. doi: 10.3389/fpls.2022.848474 (PMC9121102; doi:10.3389/fpls.2022.848474)
Supplement: Supplementary file 4 [file Data_Sheet_4.zip › Sit-miR319_Seita.2G349300.1_3579_TPlot.pdf]

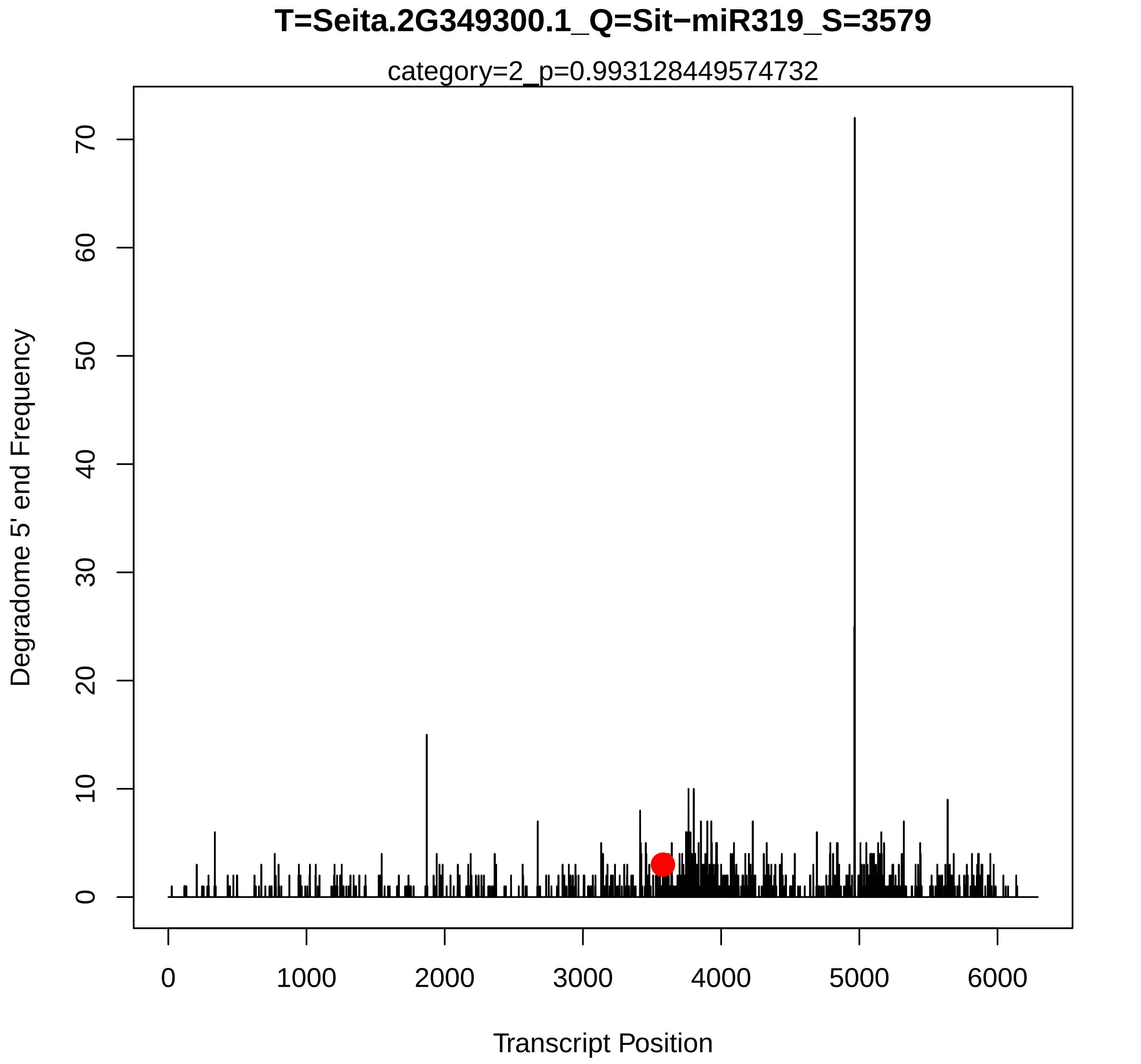
T=Seita.2G349300.1_Q=Sit−miR319_S=3579
category=2_p=0.993128449574732
Degradome 5' end Frequency
Transcript Position
0
10
20
30
40
50
60
70
0
1000
2000
3000
4000
5000
6000
